# Special Issue on the State-of-the-Art Optical Properties and Applications of Metallic Nanostructures in Asia

**DOI:** 10.3390/nano14040322

**Published:** 2024-02-06

**Authors:** Hai-Pang Chiang

**Affiliations:** Department of Optoelectronics and Materials Technology, National Taiwan Ocean University, Keelung 20224, Taiwan; hpchiang@mail.ntou.edu.tw

With developments in nanofabrication technology, the optical properties and applications of metallic nanostructures have attracted increased research interest in recent years. Top-down and bottom-up nanotechnologies have been employed to fabricate metallic nanostructures with specific optical properties. Optical excitation of the surface plasmons existing in these metallic nanostructures has given rise to brand new phenomena such as surface-enhanced Raman scattering (SERS), metal-enhanced fluorescence (MEF), Fano resonance, plasmonic photocatalysis, and metamaterials. The fundamental research and practical applications of the above optical phenomena have been widespread in the research fields of physics, chemistry, biology, and engineering. Therefore, the main focus of this Special Issue is to present the recent advances in newly developed state-of-the-art metallic nanostructures possessing the special optical properties and applications mentioned above in the fields of nanophotonics, nanobiophotonics, biophysics, and nanoengineering.

The first paper, authored by Z. Zhang, G. Wang, W. Li, L. Zhang, B. Guo, L. Ding, and X. Li, reported a novel nano-β-FeOOH/Fe_3_O_4_/biochar composite with enhanced photocatalytic performance and superparamagnetism successfully fabricated via an environmentally friendly one-step method [1]. This work highlights the enhanced photocatalytic performance of the β-FeOOH/Fe_3_O_4_/biochar material, which can be used in azo dye wastewater treatment. The second paper, authored by C. Y. Yu, Q. C. Zeng, C. J. Yu, C. Y. Han, and C. M. Wang, theoretically analyzed the phase modulation ability of a dielectric Pancharatnam–Berry (PB) phase metasurface, consisting of nanofins [2]. This design rule can be utilized to optimize the efficiency of phase-type meta-devices, such as meta-deflectors and metalenses. The third paper, authored by J. Y. Lee, C. M. Wang, C. L. Chi, S. R. Wu, Y. X. Lin, M. K. Wei, and C. H. Lin, proposed the use of an inorganic polymer composite film as an effective radiative cooling device [3]. An enhanced Seebeck effect was observed, and the corresponding output current could be enhanced 1.67-fold via photonic-assisted radiative cooling. The fourth paper, authored by J. W. Liaw, C. Y. Kuo, and S. W. Tsai, studied the effect of quasi-spherical gold nanoparticles (GNPs) on the generation of reactive oxygen species (ROS) to cause cell damage, as irradiated by a two-photon laser [4]. This study may pave the way for the use of GNPs as a photosensitized therapeutic agent for two-photon photodynamic therapy in tumor treatment. The fifth paper, authored by Y. F. Chou Chau, C. T. Chou Chao, S. Z. B. H. Jumat, M. R. R. Kooh, R. Thotagamuge, C. M. Lim, and H. P. Chiang, proposed a multiple-mode Fano-resonance-based refractive index sensor with high sensitivity; a rarely investigated structure [5]. The designed sensing structure can detect a material’s refractive index from a wide range of gases, liquids, and biomaterials (e.g., hemoglobin concentration). The sixth paper, authored by C. T. Chou Chao, Y. F. Chou Chau, S. H. Chen, H. J. Huang, C. M. Lim, M. R. R. Kooh, R. Thotagamuge, and H. P. Chiang, proposed a compact plasmonic metal–insulator–metal pressure sensor comprising a bus waveguide and a resonator, including one horizontal slot and several stubs [6]. The obtained sensitivity was increased 23.32-fold compared to the highest one reported in the literature. The modeled design paves a promising path for applications in the nanophotonic field.

The seventh paper, authored by J. C. Chen, Y. T. Chu, S. H. Chang, Y. T. Chuang, and C. L. Huang, studied plasmon-mediated shape transformation from quasi-spherical silver nanoparticles (AgNPs) to silver nanoprisms (AgNPrs) and decahedral silver nanoparticles (D-AgNPs) under irradiation from blue LEDs (λ = 456 ± 12 nm, 80 mW/cm^2^) at temperatures ranging between 60, 40, 30, 20, 10, and 0 °C [7]. The surface-enhanced Raman spectra of CV in AgNP colloids showed that D-AgNP colloids have better SERS enhancement factors than AgNPrs. The eighth paper, authored by S. Y. Yu, C. H. Tu, J. W. Liaw, and M. K. Kuo, studied the initiated plasmonic nanobubbles and the follow-up microbubbles in a gold nanorod (GNR) colloidal solution induced by a pulsed laser [8]. The advantage of a dilute GNR colloid facilitating laser-induced microbubbles in the NIR range of the bio-optical window could make biomedical applications possible. This study may provide insight into the relationship between plasmonic nanobubbles and the triggered microbubbles. The ninth paper, authored by J. Wang, Z. Li, and W. Liu, rigorously analyzed and systematically designed a double-layer metal superlens to improve subwavelength imaging ability [9]. This work provided sound theoretical analysis and a systematic design approach to a double-layer metal superlens for nearfield subwavelength imaging, such as fluorescent micro/nanoscopy or plasmonic nanolithography. The tenth paper, authored by W. T. Chen, T.-Y. Yen, Y. H. Hung, and K. Y. Lo, perform an anisotropic reflective second harmonic generation (SHG) measurement to demonstrate the sensitivity of SHG to phosphorus (P) concentrations within the range of 2.5 × 10^17^ to 1.6 × 10^20^ atoms/cm^3^ [10]. Combined anisotropic reflective SHG (Ani-RSHG) and the simplified bond–hyperpolarizability model (SBHM) can analyze the crystal structures of doped ultrathin films and provide a non-destructive nanophotonic method of in-line inspection. The last paper of this Special Issue, authored by J. Honda, K. Sugawa, H. Tahara, and J. Otsuki, is a review article focused on the use of localized surface plasmon (LSP) resonance of metal nanostructures to enhance the performance of triplet–triplet annihilation (TTA-UC) systems and explores their potential applications [11]. Specific examples of studies in which enhancements in upconverted emission have significantly improved the performance of photocatalysts under both sunlight and indoor lighting have been reported.

## Data Availability

No new data were created.
